# An easy-to-use and versatile method for building cell-laden microfibres

**DOI:** 10.1038/srep33328

**Published:** 2016-09-12

**Authors:** Jérome Kalisky, Jérémie Raso, Claire Rigothier, Murielle Rémy, Robin Siadous, Reine Bareille, Jean-Christophe Fricain, Joëlle Amedée-Vilamitjana, Hugo Oliveira, Raphaël Devillard

**Affiliations:** 1 University of Bordeaux, Tissue Bioengineering, U1026, F-33076 Bordeaux, France; 2 Inserm, Tissue Bioengineering, U1026, F-33076 Bordeaux, France; 3 CHU Bordeaux, Service de Néphrologie Transplantation Dialyse, F-33076 Bordeaux, France; 4 CHU Bordeaux, Services d’Odontologie et de Santé Buccale, F-33076 Bordeaux, France

## Abstract

Fibre-shaped materials are useful for creating different functional three-dimensional (3D) structures that could mimic complex tissues. Several methods (e.g. extrusion, laminar flow or electrospinning) have been proposed for building hydrogel microfibres, with distinctive cell types and with different degrees of complexity. However, these methods require numerous protocol adaptations in order to achieve fibre fabricating and lack the ability to control microfibre alignment. Here, we present a simple method for the production of microfibers, based on a core shell approach, composed of calcium alginate and type I collagen. The process presented here allows the removal of the calcium alginate shell, after only 24 hours of culture, leading to stable and reproducible fibre shaped cellular constructs. With time of culture cells show to distribute preferentially to the surface of the fibre and display a uniform cellular orientation. Moreover, when cultured inside the fibres, murine bone marrow mesenchymal stem cells show the capacity to differentiate towards the osteoblastic lineage, under non-osteoinductive culture conditions. This work establishes a novel method for cellular fibre fabrication that due to its inherent simplicity can be easily upscaled and applied to other cell types.

Many *in vivo* tissues are composed of microscale alignments of cells, associated or not with extracellular matrix (ECM) elements (*e.g.* microcapillaries, nerve network and muscle fibres). Diverse microfabrication methods have been proposed in an effort to produce substrates with different sizes and shapes, as to promote tissue-specific cell organization and allowing to mimic the structure of tissues. In this sense fibre-shaped materials are useful in creating different functional three-dimensional (3D) structures that can facilitate the assembly with other constructs in order to produce complex functional tissues. In contrast with classical top-down tissue engineering approaches, the fibre construct, bottom-up, approach has several advantages: (A) It is simpler, from a engineering perspective, to associate different fibre structures in order to assemble the final construct. (B) This design allows the exchange of oxygen, nutrients and cellular byproducts by cells encapsulated inside the fibres and the external media, usually limited to 150–200 μm by passive diffusion. In contrast, bulkier material approaches have shown limitations regarding cell metabolism and survival, both at the *in vitro* maturation phase and upon *in vivo* implantation.

Different strategies, with different degrees of complexity, and with the use of distinct cell sources, have been proposed in order to fabricate cellularized hydrogel microfibers, i.e. extrusion^1^, laminar flow^2^, electrospinning^3^, extrusion^4^ or multi-interfacial polyelectrolyte complexation (MIPC)^5^. These strategies, focused on tissue engineering applications, used different hydrogels and polymers^6^ and where laminar flow was the most common method for microfiber fabrication^7^. In this approach, cells are usually dispersed within hydrogels, such as calcium alginate^8^, a mix of alginate and collagen^9^, polyurethane^10^ or Poly(lactic-co-glycolic acid) (PLA-PGA)^11^. Compared with extrusion methods, using a pipette or a microfabricated nozzle, the laminar flow method allows a precise control of the fibre diameter, by adjusting the microfluidic channel dimensions or the flow rate. However, this method requires the establishment of microfluidic chambers, specific pumps and numerous protocol adjustments in order to achieve fibre fabrication.

Alginate, in spite of being one of the most common polymers used in fibre fabrication, it has shown to provide a weak interface with endothelial cells, smooth muscle cells, hepatocytes and kidney cells, upon encapsulation^12^. On the other side, collagen fibres have shown to support myocyte, neural cells or endothelial cell morphology and function^6^. In this sense, heterogenous core-shell fibres have also emerged, where cells were incorporated in a core collagen fiber inside a calcium alginate outer shell^13^. Nonetheless, this approach relies on the removal of the alginate outer shell by enzymatic degradation, using alginate lyase^13^. Another challenge in microfibre technology is to maintain fibre integrity, while cells proliferate and produce extracellular matrix, what may induce fibre shrinkage, the formation of discontinuous cellularized fiber segments and a possible fibre aggregation with time of culture.

Here, we present a new ready-to-use method for the production of microfibers, using a core shell approach, composed of calcium alginate shell and a cellularized type I collagen core. This process doesn’t require specific devices or a high cell density. Another added value to this new approach concerns the easy removal of the calcium alginate hydrogel, without enzymatic degradation, after only 24 hrs of culture, and while preventing fibre aggregation up to two weeks of culture. Additionally, this approach enables an easy adaptation to novel shape design, while allowing a tight control over fibre reproducibility.

## Results

**Figure 1:**
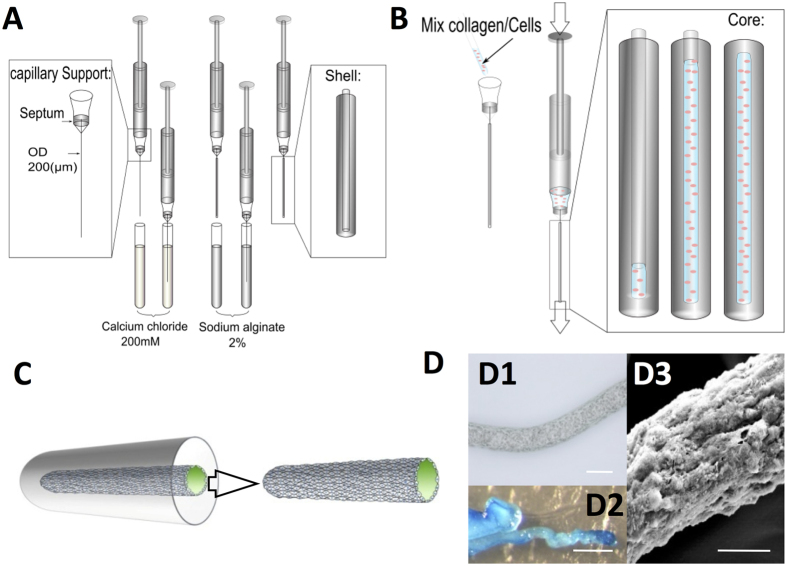
Fabrication procedure of the core - shell fibres. (**A**) Production of the shell using successive alginate coatings. (**B**) Production of the core by the substitution of the capillary with collagen/cell suspension. (**C**) Ability to separate the core from the shell after 24 h of culture. **(D1)** Representative contrast phase microscopy of a fibre at day 4 (scale = 150 μm). (**D2)** Representative coloration with methyl blue of a fibre at day 4 (scale = 500 μm). (**D3**) Representative scanning Electron Microscopy of a fibre at day 4 (scale = 50 μm).

The method for cell fibre fabrication is presented in Fig. 1 and in the supplementary material (Supplementary movie S1 for detailed fabrication process). This fabrication approach consists on three steps; the first one concerns the preparation of the calcium alginate shell (Fig. 1A). Then a core of cells encapsulated inside collagen, of 200 μm diameter, is introduced within the calcium alginate shell (Fig. 1B). The third step consists on the removal of the calcium alginate shell, after 24 hours of culture, leading to a stable and reproducible fibre shaped cellular construct (Fig. 1C and Supplementary Figure S1). As observed in Fig. 1D1,1D2, with the macroscopic evaluation and the methyl blue collagen staining of the fibres after 1 day of culture, respectively, the obtained fibres are homogenous in size and maintain their shape after the calcium-alginate shell removal. Evaluation by scanning Electron Microscopy (SEM), performed after 24 hrs of culture, show cells embedded in the collagen matrix (Fig. 1D3). By using this method, we generated homogenous fibres with controllable diameter, 119 ± 11 µm (n = 5), and around 3 cm of length.

**Figure 2:**
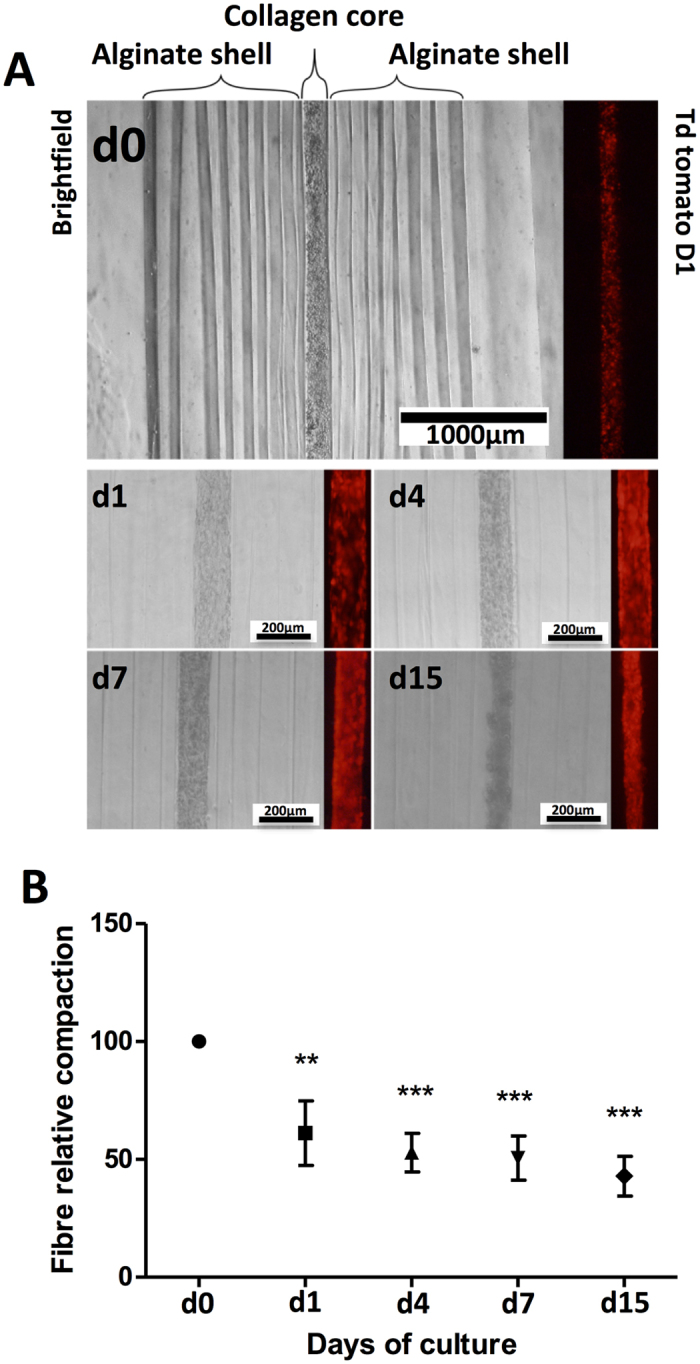
Analysis of the compaction of the microfibers containing D1-tdTomato cells, with time of culture. (**A)** Microscopic evaluation of fiber compaction at days 0 (d0), 1 (d1), 4 (d4), 7 (d7) and 15 (d15). (**B)** Fibre compaction quantification evaluated by the measurement of the fibre diameter, with time of culture, at days 0 (d0), 1 (d1), 4 (d4), 7 (d7) and 15 (d15) (Average ± SD, n = 6).

Then, we evaluated the behavior of cells encapsulated within the core gel. For this purpose we used the D1 cell line, a multipotent mouse bone marrow stromal precursor cell line, as a cellular culture model. Stable D1-tdTomato cells were first generated by lentivus infection^14^, allowing the direct evaluation of cell arrangement and organization, within the fibres, with time of culture. As observed in Fig. 2A,B, cells were well dispersed in the collagen core and induce the contraction of the fibre, with time of culture from days 0 (d0) to 15 (d15) (Fig. 2B).

**Figure 3:**
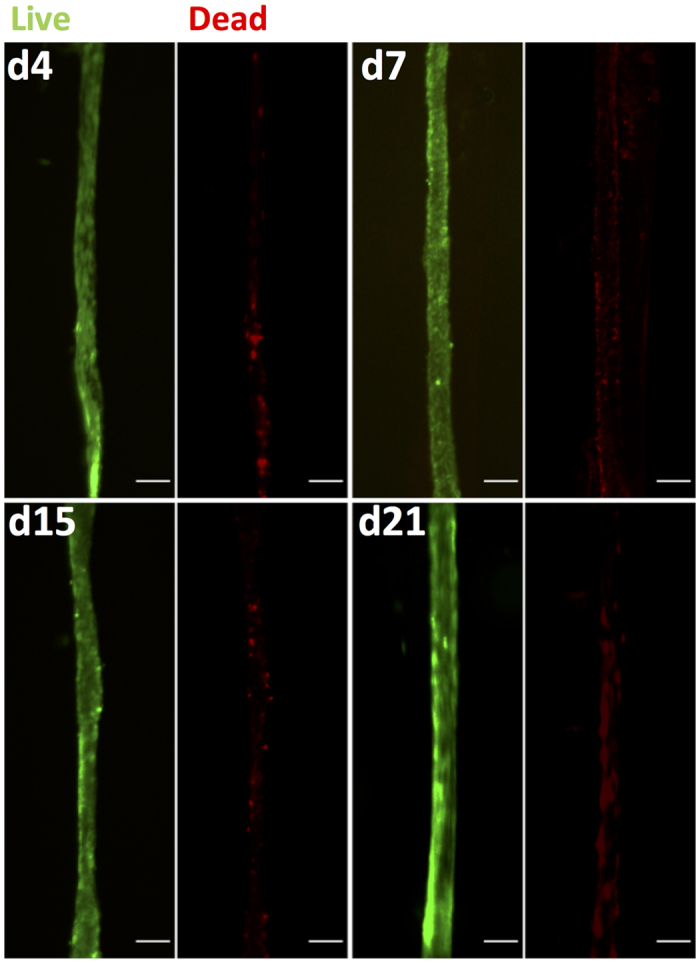
Cell viability evaluation using the Live/Dead assay. Live and dead cells were stained with calcein (green) and ethidium homodimer-1 (red), respectively, at days 4 (d4), 7 (d7), 15 (d15) and 21 (d21) (scale = 200 μm).

In order to assess the viability of the encapsulated cells within the collagen gel we performed a Live/Dead^®^ assay, capable to discriminate between viable and non-viable cells. As observed in Fig. 3 only few red cells could be observed with time of culture from days 4 (d4) to 21 (d21).

**Figure 4:**
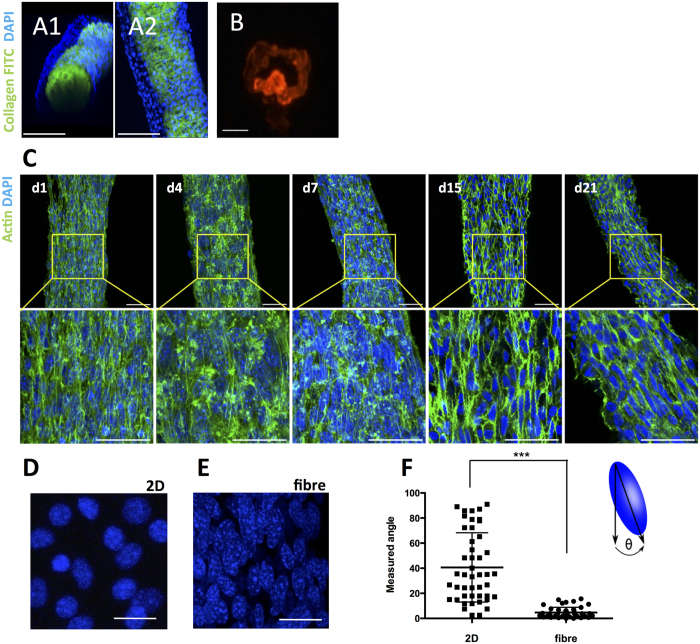
Cell distribution within the microfibres. (**A1,A2)** Representative 3D confocal microscopy reconstruction images of fibres containing D1 cells. FITC-Collagen I was used for the fabrication of the fibres (green) and nuclei of the D1 cells were stained with DAPI (blue) (scale = 100 μm). (**B)** Representative fluorescence microscopy cryosection of the fibres seeded with D1-td-tomato cells, after 1 day of culture (scale = 50 μm). (**C)** Analysis of the actin cytoskeleton of D1 cells within the fibres at Days 1 (d1), 4 (d4), 7 (d7), 15 (d15) and 21 (d21). F-actin (green) and nuclei (blue) were stained with phalloidin and DAPI, respectively (Scale = 50 μm). Representative images of DAPI stained D1 cell nucleus, cultured in 2D (**D)** or 3D (inside the fibres) (**E)** after 7 days of culture (Scale = 50 μm). (**F)** Image analysis quantification of the cell nucleus orientation angle, in relation to a vertical axis or the fibre axis, for D1 cells cultured in 2D or inside the fibres respectively for 7 days.

We then evaluated the distribution of cells, with time of culture, within the collagen matrix. For that we used FITC-conjugated type I collagen and labeled the cells using DAPI staining. As seen in Fig. 4, after one day of culture D1 cells, stained with DAPI (Fig. 4A1,A2) are mainly distributed in the periphery of the collagen fibres. These results were confirmed in the transversal cryosections of the fibres, constructed with D1-TdTomato cells embedded in collagen fibre core (Fig. 4B). The subsequent F-actin and DAPI staining (Fig. 4C) shows well oriented actin filaments with time of culture from days 1 to 21 (d1–d21, Fig. 4C).

In order to quantify this orientation, image analysis was used to assess the angle of the cell nucleus in relation to the axis of the fibre. D1 cells cultured in 2D conditions showed a round nucleus appearance (Fig. 4D, 7 days), in contrast with D1 cells cultured inside the fibres (Fig. 4E, 7 days) that show an elongated orientation. The quantification of the cell nucleus orientation angle in relation to a vertical axis, meaning in relation with the fibre orientation in the case of the 3D cultured cells, shows a significant decrease of the average angle, indicative of a tight orientation of the cells (Fig. 4F). Additionally, we also performed different time points of shell removal (*i.e*. 24 and 48 hours) and evaluated the cellular orientation after additional 24 hrs of culture. In these two early time points no significant differences could be observed, in relation with cells cultured in 2D conditions (angle values were 53 ± 19 and 52 ± 20, respectively, Average ± SD, n = 50).

**Figure 5:**
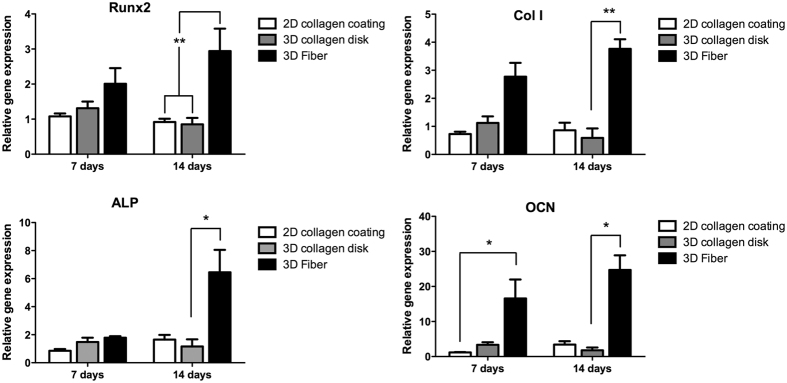
Quantitative analysis of osteoblastic-specific gene expression. Quantitative gene expression of Runx2, Col I, ALP and OCN in D1 cells cultured in 2D conditions (wells coated with collagen type I), in 3D conditions in collagen type I disks or in 3D collagen fibres, at 7 and 14 days (Average ± SD, n = 3). * and ** denotes p < 0.05 and p < 0.01, respectively.

The differentiation of D1 cells, towards the osteoblastic lineage, was followed inside the fibres by means of qPCR evaluation of Runx2, collagen type I (Col I), alkaline phosphatase (ALP) and osteocalcine (OCN) at both 7 and 14 days (Fig. 5). Significant increase on gene expression was observed for Runx2, Col I and ALP at 14 days and for OCN at both time points, in relation with cells cultured in 2D conditions or 3D inside collagen disks (see Fig. 5).

**Figure 6:**
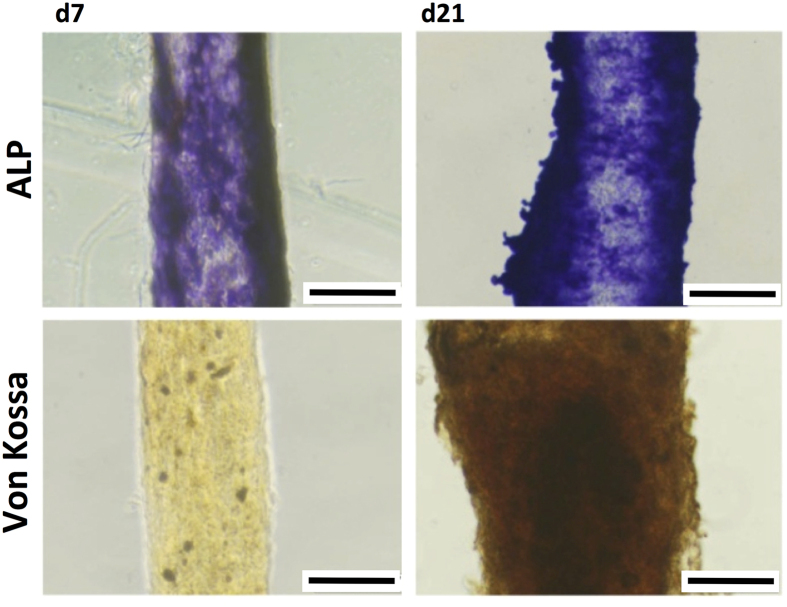
Alkaline phosphatase staining and mineralization evaluation of D1 cells encapsulated inside the fibres. Alkaline phosphatase and Von kossa staining at days 7 (d7) and 21 (d21) of culture in a non -osteoinductive medium (scale = 100 μm).

These results were confirmed using the Alkaline Phosphatase (ALP) and by Von Kossa stainnings, from days 4 (d4) to 21 (d21) in the absence of osteogenic media (Fig. 6). One can observe that, even in basal media, D1 cells are able to differentiate towards the osteoblastic phenotype, as observed by the intense ALP staining and by the deposition of a mineralized matrix (Von Kossa staining) after 21 days of culture (Fig. 6).

## Discussion

Here, we report a novel and simple method to obtain cell-laden microfibers, with controllable diameter, compatible with a wide range of cell types and without the need for specific apparatus or enzymatic degradation of the shell. The method is based on the creation of an alginate shell that after gelation with a calcium solution serves as a mold for a cellularized collagen suspension, that after only 24 hrs of maturation enables to form a gel fibre able to be manipulated. The ensemble of the presented results shows that this easy-to-use method allows the production of cell fibres, able to support cell growth and differentiation for at least three weeks of culture.

Compared to the other methods, such as the one described by Onoe *et al*., based on a coaxial microfluidic system^13^, our method mainly differs by the absence of specific equipment and materials in order to build reproducible and functional cell-fibres. In the same way, Kang *et al*.^15^ have presented a spinning method that uses a microfluidic chip combined with a digital fluid controller system. Additionally, the same authors proposed a method of continuously fabricating microfibers with size-tunable grooved microstructures using a microfluidic system^16^. In another study Sakai *et al*.^17^ demonstrated that they can successfully fabricate a tubular construct with cells embedded in a collagen gel, using oxidized alginate - cross linked calcium alginate/gelatin fibres. However, it still requires a step of degradation with alginate lyase, what can imply toxicity to the encapsulated cells and compromise the construct use in subsequent applications.

As expected, by our method we can manually remove the calcium alginate shell without enzymatic digestion of the calcium alginate shell. This possibility ensures less damage to cells, while allowing a complete elimination of alginate prior to tissue maturation, differentiation processes or implantation in an experimental model.

Different hydrogels can be advantageously used for microfibre production^13^. As example, chitosan-based microfiber cell-laden were evaluated as scaffold in order to create liver tissue constructs^18^. Among other natural polymers, collagen offers many advantages in a perspective of tissue engineering and regenerative medicine. The choice of this natural hydrogel is based on the lower inflammatory response, its biodegradability, and weak antigenicity and the possibility for cells to modify their microenvironment by the production of extracellular matrix proteins^19^. The proposed approach for fibre design provides both sufficient mechanical support, by the alginate shell, and sustainable cell growth conditions, given by the collagen core.

In line with previous reports, here we show that cellularized collagen fibers contract with time of culture. This effect, based on the interaction between the encapsulated cells and the fibrilar structure of collagen, leads to a reorganization of the collagen matrix allowing the modulation of the forces applied by and to the cells^20^. This dynamic interplay between cells and the matrix is crucial in order to allow the production of extracellular matrix and sustain tissue maturation. Here, we focused on the use of a mouse bone marrow mesenchymal stem cell source (D1 cell line) and show that in the absence of osteogenic media cells show to differentiate into the osteoblastic lineage. Previous reports support this observation, where mesenchymal stem and osteoblastic cell culture within collagen gels showed to generate internal loads, inside the gels, leading to gel contraction and differentiation towards the osteoblastic lineage^21^,^22^,^23^.

In this work we focused on a bone tissue engineering approach, nonetheless, the proposed methodology can be easily adapted to other tissue engineering and/or drug delivery applications. Due to its inherent simplicity, fibre diameter and geometry can be easily modified, by changing the type of capillaries used, allowing adjusting to different cellular requirements and end use applications. Also, we show that with the described methodology cells migrate to the periphery of the microfibre and display a uniform alignment along fibre axis, in the absence of fluid flow. This micro-engineering approach may encourage innovation of a wide application areas such as neuron cell alignment for nerve regeneration, or the building of circumferentially oriented smooth muscle like tissue^24^. In addition, several combinations with specific cell-laden fibres can be proposed for vascularized bone tissue engineering^25^, as well as for the co-culture of two cell types within the same micro-fibres, promoting cell communication, crucial for the building of a functional tissue. Indeed, we are currently exploring the capacity of this fibre arrangement to orient cells, without the use of laminar flow, in the fabrication of microvessel like structures using human endothelial progenitor cells. These studies will enable to widen the horizon of this technological approach to other applications. Additionally, conscious of the inherent variability associated with human manipulation we are envisaging to adapt the described method using multiple capillary units, associated with a mechanic operated injection system, what will allow to increase the volume of fibers produced and reduce human related variability.

In conclusion, here we show a simple, reproducible and straightforward production method to obtain cellularized fibres, which sustain cell function. This approach can find use on the production of cellularized fibres that can mimic the morphological structure of *in vivo* tissues, such as nerves bundles, blood vessels or muscle fibres.

## Methods

Type I Collagen was purchased from BD Biosciences (Collagen Type I, obtained from rat tail, Bedford, US). Calcium Chloride (CC), sodium hydroxide (NaOH) and ethanol (EtOH) were purchased from Sigma (Saint Quentin Fallavier, France). Sodium alginate Protanal LF-10/60 was purchased from (FMC Biopolymer, Drammen, Norway). Phosphate Buffer Saline 0.1 M pH 7 (PBS) was from Gibco (Life Technology SAS, Saint Aubin, France). D1 cell line was purchased from ATCC (LGC Standards, Molsheim, France).

### Cell culture

Prior use, D1 cell line were cultured in 2D conditions, in 150 cm^2^ culture flasks, with Dulbecco’s Modified Eagle Medium (DMEM, Gibco, Life Technologies) supplemented with 10% (v/v) foetal calf serum (FCS) (Lonza, Levallois Perret, France) at 37 °C and with 5% CO_2_.

### Microfibre biofabrication

The different steps of the biofabrication were summarized in Fig. 1 and on the supplementary movie 1 (supplementary data).

The capillary (Paradigm Optics, Vancouver, USA) with a diameter ID/OD respectively of 200/300 μm and combined with a suitable septum (Sigma-Aldrich, Saint Quentin Fallavier, France), of 5 mm diameter, was introduced into the needle 18 G (Dutscher, Brumath, France).

Sodium alginate (SA) Protanal LF-10/60 (FMC Biopolymer, Drammen, Norway) was prepared at 2% (w/v) in PBS and cross-linked with Calcium Chloride (CC) (Sigma, Saint Quentin Fallavier, France) dissolved in deionized water at 200 mM. The capillary support was successively dipped into the CC then SA, until a diameter shell of 7 mm was obtained. Samples were then transferred into petri dishes containing PBS, prior to the supplementation with the collagen solution.

Type I Collagen (Rat Collagen Type I, Bedford, US) was prepared at 5 mg/mL, according to the manufacturer’s instructions. Confluent D1 cells were collected from the culture flask by trypsin treatment (trypsin solution at 0.1 mg/mL in EDTA 0.065 mg/mL) and were mixed with the neutralized collagen to obtain a final cell density of 50 × 10^6^ cells/mL.

This mix was introduced into the syringe, equipped with the capillary support previously prepared, and introduced within the shell of alginate to perform the core. After washing with PBS, the microfibres were then cultured in 6 well plates at 37 °C and 5% CO_2_, with DMEM supplemented with 10% (v/v) FCS. This non-osteoinductive medium was replaced every two days.

The core could then be mechanically removed from the shell after one day of culture, as indicated in Fig. 1 and on the video (supplementary data).

Controls were performed without the addition of D1 cells in the fibres. Two-dimensional cultures of D1 cells on plastic dishes were performed in non-osteoinductive as well.

### Cell viability evaluation

A Live-Dead assay (Thermofisher Scientific) was performed according to the manufacturer’s instructions. The Live-Dead Cell Staining Kit provides the ready-to-use reagents for convenient discrimination between live and dead cells. The kit utilizes Live-Dye, a cell-permeable green fluorescent dye (Ex/Em = 488/518 nm), to stain live cells. Dead cells can be easily stained by ethidium homodimer-1 a cell non-permeable red fluorescent dye (Ex/Em = 488/615). Stained live and dead cells within the fibres were visualized by fluorescence microscopy (Axiovert microscope, 25 CFL, Zeiss, Germany).

### D1 cells actin staining

After 1 and 21 days of culture, samples were washed twice with PBS and fixed with 4% paraformaldehyde (Sigma-Aldrich), in PBS, for 45 minutes at 4 °C. Core and shell samples were then permeabilized with 0.1% (v/v) Triton X-100, in PBS, for 15 minutes, followed by blocking with 1% (w/v) bovine serum albumin (BSA), in PBS, for 30 min at room temperature.

Samples were then incubated with Alexa fluor 488 Phalloidin (Invitrogen, Life Technologies), diluted 1/40 in PBS with 1% BSA, for 1 hour at 37 °C. After washing two times with PBS, containing 0.05% Tween 20, samples were incubated with 4′,6′-diamidino-2-phenylindole dihydrochloride (DAPI, Invitrogen, Life Technologies) at 1 μg/mL, in PBS, for 10 min at room temperature. Finally, fibres were mounted in Fluoromount G mounting media (Southernbiotech, USA), on coverslips, before analysis with a confocal laser-scanning microscope (SPE, Leica Microsystems).

### D1 cells orientation determination

In order to determine cell orientation inside the fibres, samples at 7 days of culture were fixed, stained with DAPI and imaged by confocal microscopy, as previously described. Using Image J (1.47) software we defined for each individual nucleus a ROI, fitted an ellipse, and determined the angle between the major axis of the ellipse and the axis of orientation of the fibre or a vertical axis for 3D or 2D cultured cells, respectively.

### Quantitative real-time polymerase chain reaction (qPCR)

As means to evaluate the capacity of D1 cells to undergo differentiation towards the osteoblastic lineage under different culture conditions we tested three different culture conditions. D1 cells were cultivated during 7 and 14 days in: 2D conditions, over collagen type 1 (0.1 mg/mL) coated TCPS plastic at 10 000 cells/cm^2^; in 3D conditions in collagen gel disks or inside the fibres (collagen type I at 5 mg/mL, as before) and at a cell density of 10 M cells/mL, for both conditions. At defined time points total RNA was extracted using the RNeasy Total RNA kit (Qiagen), as indicated by the manufacturer, and 1 μg was used as the template for single-strand cDNA synthesis, using the Superscript system (cat#11904-018) (Invitrogen, Carlsbad, CA, USA). cDNA diluted at a 1:80 ratio was loaded onto a 96-well plate. Real-time PCR amplification was performed using the Takyon no-rox Supermix (Eurogentec, France). Primers of the ubiquitary protein glyceraldehyde-3-phosphate dehydrogenase (GAPDH; forward 5′-AGGAGCGAGACCCCACTAAC-3′, reverse 5′- TCACAAACATGGGGGCATCG-3′), Cbfa1/runx2 (forward 5′-CCTCAGTGATTTAGGGCGCA-3′, reverse 5′-TGGTGCAGAGTTCAGGGAGG-3′), ALP (forward 5′-CCGAATCCTTAAGGGCCAGC-3′, reverse 5′-ACCTCATTGCCCTGAGTGGT-3′), Col 1 (forward 5′-CGAGTCACACCGGAACTTGG-3′, reverse 5′-TGGGAGGGAACCAGATTGGG-3′) and OCN (forward 5′-ACCTCACAGATGCCAAGCCC-3′, reverse 5′-AGCGCCGGAGTCTGTTCAC-3′), were used at a final concentration of 500 nM. Data were analyzed using iCycler IQ software and compared by the ΔΔCT method. Q-PCR was performed in triplicate. Data was normalized to GAPDH mRNA expression for each condition and was quantified relative to Runx2, Col I, ALP and OCN gene expression in D1 cells cultured in 2D conditions after 7 days, which was standardized to 1.

### Alkaline phosphatase staining

ALP staining of D1 cells, encapsulated within the fibres, was performed using the Alkaline Phosphatase kit 86 C-1KT (with fast blue BB) (Sigma-Aldrich), as described in the manufacturer’s instructions. Briefly, core and shell samples were incubated with Fast Blue RR salt and Naphtol AS-MX Phosphate Alkaline solution 0.25% (Sigma-Aldrich) during 30 min at room temperature, in the dark. Samples were analyzed using a stereo microscope (MZ10F, Leica Microsystems, Germany).

### Von Kossa staining

Deposition of calcium within the fibres was evaluated using the Von Kossa staining. Briefly, fibres deprived of the alginate shell were incubated with 2.5% (w/v) silver nitrate for 30 min at room temperature, then incubated in distilled water for 10 min and finally in 30% formol/sodium carbonate for 2 to 5 min. The samples were analyzed using a stereo microscope (MZ10F, Leica Microsystems, Germany).

### Statistical analysis

Using the Graphpad Prism 5.0 software, the D’Agostino and Pearson omnibus normality test was used in order to verify if data conformed to a Gaussian distribution. A statistical significant difference between two groups was determined by the non-parametric Mann Whitney test. A difference between more than two groups was analyzed by the non-parametric Kruskal-Wallis test, followed by a Dunns post-test. Results were considered statistically significant for p value lower than 0.05.

## Additional Information

**How to cite this article**: Kalisky, J. *et al*. An easy-to-use and versatile method for building cell-laden microfibres. *Sci. Rep.* **6**, 33328; doi: 10.1038/srep33328 (2016).

## Supplementary Material

Supplementary Information

Supplementary Movie S1
