# Supplementary material for: An easy-to-use and versatile method for building cell-laden microfibres
Source: Sci Rep. 2016 Sep 12;6:33328. doi: 10.1038/srep33328 (PMC5018858; doi:10.1038/srep33328)
Supplement: Supplementary Information [file srep33328-s1.doc]

Supplementary Information

**An easy-to-use and versatile method for building cell-laden microfibres**

Jérome Kalisky, Jérémie Raso, Claire Rigothier, Murielle Rémy, Robin Siadous, Reine Bareille, Jean-Christophe Fricain, Joëlle Amedée-Vilamitjana, Hugo Oliveira, Raphaël Devillard.

**Supplementary Data**

**Supplementary Movie S1**: Movie depicting the protocol for fibre production.
